# Primary care practices’ ability to predict future risk of expenditures and hospitalization using risk stratification and segmentation

**DOI:** 10.1186/s12911-021-01455-4

**Published:** 2021-03-18

**Authors:** David A. Dorr, Rachel L. Ross, Deborah Cohen, Devan Kansagara, Katrina Ramsey, Bhavaya Sachdeva, Jonathan P. Weiner

**Affiliations:** 1 Department of Medical Informatics and Clinical Epidemiology, Oregon Health and Science University, 3030 SW Moody Ave, Portland, OR 97201 USA; 2 VA Portland Health Care System, Portland, OR USA; 3 Johns Hopkins University, Baltimore, MD USA

**Keywords:** Risk assessment, Chronic disease, Primary care, Care management

## Abstract

**Background:**

Patients with complex health care needs may suffer adverse outcomes from fragmented and delayed care, reducing well-being and increasing health care costs. Health reform efforts, especially those in primary care, attempt to mitigate risk of adverse outcomes by better targeting resources to those most in need. However, predicting who is susceptible to adverse outcomes, such as unplanned hospitalizations, ED visits, or other potentially avoidable expenditures, can be difficult, and providing intensive levels of resources to all patients is neither wanted nor efficient. Our objective was to understand if primary care teams can predict patient risk better than standard risk scores.

**Methods:**

Six primary care practices risk stratified their entire patient population over a 2-year period, and worked to mitigate risk for those at high risk through care management and coordination. Individual patient risk scores created by the practices were collected and compared to a common risk score (Hierarchical Condition Categories) in their ability to predict future expenditures, ED visits, and hospitalizations. Accuracy of predictions, sensitivity, positive predictive values (PPV), and c-statistics were calculated for each risk scoring type. Analyses were stratified by whether the practice used intuition alone, an algorithm alone, or adjudicated an algorithmic risk score.

**Results:**

In all, 40,342 patients were risk stratified. Practice scores had 38.6% agreement with HCC scores on identification of high-risk patients. For the 3,381 patients with reliable outcomes data, accuracy was high (0.71–0.88) but sensitivity and PPV were low (0.16–0.40). Practice-created scores had 0.02–0.14 lower sensitivity, specificity and PPV compared to HCC in prediction of outcomes. Practices using adjudication had, on average, .16 higher sensitivity.

**Conclusions:**

Practices using simple risk stratification techniques had slightly worse accuracy in predicting common outcomes than HCC, but adjudication improved prediction.

**Supplementary Information:**

The online version contains supplementary material available at 10.1186/s12911-021-01455-4.

## Introduction

### Background

Risk stratification, a process by which risks to health and well-being for a population of patients are quantified and patients are grouped by risk, was historically performed to manage expected health care expenditures. For instance, payers utilized risk scores to actuarially adjust insurance premiums or identify insured persons at high risk of poor outcomes or high expenditures [1, 2]. With the advent of new data sources, such as patient-reported outcomes, and tools, such as the electronic health record (EHR), which make these data more accessible, the process of stratifying a patient panel at the point of care in real time is possible. Doing so may help allocate limited health care resources—such as nurse care managers, social workers, behavioral specialists, pharmacists, or high-risk patient teams—who can tailor care to mitigate risks and improve such patient outcomes as emergency department (ED) visits or hospital readmissions. For a majority of patients, primary care—the place where they first seek ongoing care and where care is coordinated—may be a natural place to perform stratification [3]. For instance, a patient with new onset dementia and a patient with severe depression and coronary artery disease might both have high needs, but the approach to mitigate future adverse outcomes might be quite different.

However, current algorithms to perform risk stratification have only had moderate success in translation from insurers to primary care; this is partially due to significant variation in approach—data sources, completeness, and accuracy of data varies between insurance or claims data and the clinical data available to providers [4]. In addition, standard risk scores may be useful for risk adjustment but only have moderate predictive ability, indicating significant room for improvement [5–7]. Risk stratification is challenging, in part, due to many unmeasured or incomplete aspects of risk, including social and behavioral issues such as food insecurity or self-efficacy [8]. The translation of risk stratification from larger organizations with requisite analytic skills may be a significant barrier to their successful implementation, yet using the intuition, or human judgement, of health care professionals may improve the use of risk scores [9]; some have found better prediction using adjudication—starting with an algorithmic score, then using human judgment—in specific populations, especially when training is provided [10, 11]. However, the ability of clinical teams, especially at diverse practices, to improve risk scores and tailor care more effectively outside of highly-specialized or large health system settings remains unknown.

### Purpose

Our purpose was to use the experience of a diverse set of primary care practices—urban, rural, independent, and in smaller health systems—to see how their risk stratification process compares to that available by standard risk scores, and which approach identified patients at high risk of utilization more accurately. We used, as our study base, a natural experiment where every primary care practice was asked to risk stratify their populations, and combined data from the practice’s RS process with outcomes data to understand the predictive accuracy of their approaches. Our hypothesis was that adjudication of algorithmic risk scores would show improved performance (predicting patients with high risk of hospitalizations or ED visits) over either clinical intuition or algorithmic, machine-generated risk scores alone.

## Methods

### Study design

The study was part of a mixed methods effort to assess practices’ confidence in risk stratification approaches, and factors relating to successful adoption of these workflows. We asked each participating practice to provide the risk stratification scores or strata they had created, the specific demographic and diagnostic information about the patients they risk stratified from their EHR system, and utilization outcomes. We used these data to compare their scores to the Hierarchical Condition Categories (HCC), a standard score used for Medicare risk adjustment, and to compare both their scores and the HCC in predicting future utilization outcomes. The Institutional Review Board at Oregon Health & Science University (OHSU) approved of this study.

### Setting and participants

Primary care practices in three states—Oregon, Colorado, and Ohio—were eligible to participate in this study if they participated in one of several health reform initiatives (e.g., accountable care organization, multi-payer advanced primary care demonstrations, Comprehensive Primary Care initiative from the Centers for Medicare and Medicaid Services) where they were asked to risk stratify their patient population and then tailor the care of high needs patients based on risk. All eligible practices identified from public participation lists (N = 150) were first asked to fill out a general survey about risk stratification; those that responded and had actively risk stratified > 90% of their clinic populations were asked to submit data for the study. In the initiatives, practices were asked to use any risk stratification method they felt would best predict adverse outcomes for their populations; they were given technical assistance in the form of webinars and limited technical support, but in general, they had to implement and use their own approach. Practice risk tools included original algorithms and versions of the American Academy of Family Physicians (AAFP) patient risk rubric. Some practices allowed providers (MD, DO, NP, PA) to use their own clinical intuition or adjudicate scores, while others also allowed care team members to adjust scores.

### Data collection and measures

#### Practice level measures

An online survey was developed to understand the practices' approach to and perception of risk stratification; its development and results are described elsewhere [9], but consisted of questions submitted via REDCap [12] about practice demographics, risk stratification approach development, and their assessment of its value for the care of patients. We used size, location (urban vs. rural), and ownership (independent vs. health system) for practice characteristics. For their risk stratification approach, we classified the practices as using clinical intuition alone to assign a risk tier versus those using an algorithm and changing, or adjudicating, the score. For perceived confidence of the process, we used a summary factor for each practice on whether their approach was ideal, correct, and generated confidence; low was < 50% agreement about all 3, high was > 50%.

#### Patient-level data

For each practice, a trained informatician and data architect used a structured data extraction approach of active patients (persons seen within the last 2 years) from their EHR and other HIT systems. Besides the risk stratification scores stored by the practice, the team extracted demographic, diagnosis, and utilization information from the practices. For outcomes, we worked with practices to assess and compile information about persons on whom they had comprehensive information on expenditure and utilization as part of their initiative. If they received reliable information from payers about the outcomes of their patients, we helped them integrate this into the data feed. We de-identified the information and all analyses were performed on de-identified information.

### Key measures and outcomes

Key measures were the tier (low, medium, high, and very high) of both practice risk score and a standard risk score, the Hierarchical Condition Categories. The practice-based risk scores varied in form, but all provided strata or tiers for 4 levels of risk: low, moderate, high, and very high. The HCC score is a standardized measure of the risk for future utilization (range 0.25–5.7); we implemented this on the data extracted from practices, using age, sex, and diagnosis codes. Practices generally had access only to data for their own visits. Based on each practice’s population size, we assigned tier cut-points in the HCC scores to generate similar sized tiers and allow for comparisons with the practice’s risk score calculations. Outcomes, which were extracted from payer reports, were total reported expenditures, counts of reported hospitalizations, and counts of ED visits for each person in the year before and after the risk stratification date (where date of risk stratification is considered day 0), using cut-points for each that attempted to match the percent of patients stratified at high risk with the same percentage who had high expenditure or utilization outcomes. The cut-points were expenditures over $30,000, 2 or more ED visits in the prior year; and 1 or more hospitalization in the prior year. These cut-points were created to capture the roughly 10% (9–14% by category) of patients who accounted for roughly 2/3 of total utilization.

### Analysis

To form the final analytic dataset, we assessed HIT data quality, retained patients with complete data, and integrated the data from the survey. With EHR extraction, data quality was a major issue and was assessed at several stages, from initial assessments of correctness and completeness by comparing descriptive statistics to final assessments of practice-based variation. Patients were retained for analysis if they were seen prior to risk stratification, had a valid practice-based risk score, and their records did not indicate death or that they left the practice during the 2-year observational period. For outcomes analysis, they needed a year of outcomes after the risk score was calculated.

We first completed descriptive analyses of the practices, their survey responses, and their patient populations. We then compared agreement on who was high risk by calculating the contingency table, overall agreement, and kappa for the HCC score highest tier compared to the practice’s highest tier. Then, we limited the analysis to those with reliable outcomes, roughly 10% of the overall sample; only Medicare and dual eligible patients had reliable outcomes. We then compared the highest tier of both HCC and practice risk scores against the cut-offs for utilization (described above), to calculate practices’ overall diagnostic performance in their classification of patients at high risk for future negative health outcomes. We calculated their sensitivity (true positives, TP)/(TP + False negatives, FN), specificity(True Negatives, TN)/(TN + False Positives, FP), positive predictive value(TP/(TP + FP), negative predictive value(TN/(TN + FN)), and overall accuracy (proportion of correctly classified cases, or (TP + TN/ ALL) for each of the outcomes, focusing on the sensitivity and positive predictive value as primary outcomes; significant differences between tests were calculated using paired-test comparison of two proportions from Moskowitz and Pepe [13]. We performed sub-analyses based on risk stratification approach—clinical intuition or adjudicated algorithm—and overall perception of approach to the predictive validity of the approach.

## Results

In all, 100 people from 37 practices filled out the initial survey and had risk stratified their population; of these, 12 contacts from 12 practices consented to participate, with 25 practices declining or not responding to the request to participate. Of these 12 practices, 3 did not store their risk stratification scores in a structured manner that included the date of risk stratification, and 3 withdrew prior to data extraction, leaving 6 practices for this analysis, all of which were located in Oregon.

Practice and patient characteristics are shown in Table 1. Of the six practices included, 4 were moderate size (a two-year unique patient panel of 10,000–20,000 patients seen), 2 were rural, and 4 were health system owned, and all were located in Oregon. Practices had a total of 40,342 eligible patients with risk stratification scores. Practices reported using one of three types of risk stratification: intuition alone (n = 1), algorithmic stratification alone (n = 1), and algorithmic stratification with adjudication (n = 4). Intuition was only completed by providers (MD, DO, NP, PAs) and adjudication was predominantly providers (N = 3) with one practice allowing care team adjudication. Within a practice, those completing the survey had high levels of agreement on the quality of their risk stratification approach. These views of quality, however, varied across practices, with three practices agreeing that their approach was strong (high) and three feeling it still was not correct or ideal (low). For the second analytic phase, 3,381 patients had reliable records of hospitalizations and ED visits; drop out was largely due to insurance turnover, practice turnover, and outcome availability. Reliability was determined by consistent utilization records the year prior to and after the risk stratification score was calculated.

**Table 1:** Description of included practices and approach

Practices (N = 6)	Result
Practice size	
Large (greater than 20,000 patients)	2 (33%)
Medium (10,000–20,000 patients)	4 (67%)
Practice location	
Urban	4 (83%)
Rural	2 (17%)
Practice ownership	
Health system	4 (67%)
Independent	2 (33%)
Perception of practice RS process^a^	
High (> 67% agree)	3 (50%)
Low (< 33% agree)	3 (50%)
Stratification approach	
Clinical intuition	1 (17%)
Algorithm only	1 (17%)
Adjudication	4 (67%)
Patient panels N (range)	40,342 (2,209–24,192)
% female (practice range)	57% (54–60%)
Age mean (practice range)	60.8 (56.5–72.0)
HCC scores (IQR)	0.60 (0.28–0.80)
Practice 1	0.62 (0.35–0.79)
Practice 2	0.74 (0.35–1.0)
Practice 3	0.54 (0.29–0.67)
Practice 4	0.55 (0.26–0.70)
Practice 5	0.65 (0.29–0.86)
Practice 6	0.79 (0.40–1.05)
HCC categories mean (practice range)	0.79 (0.47–1.06)
HCC conditions % (range)	
Diabetes	11% (2–19%)
Neoplasm	11% (3–12%)
Heart disease	10% (7–17%)
Psychiatric	8% (2–14%)
Lung	5% (3–9%)
Patients with outcome data available	3,381 (49–1,629)
Outcome rate % (range)	
At least one ED visit	30% (25–59%)
At least two ED visits	12% (9–36%)
One or more hospitalization	14% (11–33%)
Expenditures ≥ $ 30,000	9% (6–22%)

^a^Percent agreement with measures of risk score correctness and overall confidence in stratification approach; no medium levels of agreement were seen (34–66%)

**Fig. 1:**
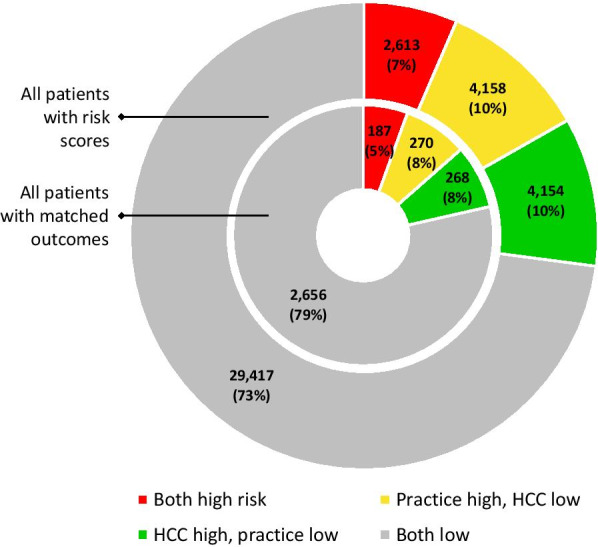
Diagnostic characteristics of practice-derived scores versus HCC scores top categories

Figure 1 demonstrates the overall performance characteristics of the practice-derived high risk category versus the standard HCC risk score. In the outside circle, all scored patients are compared; of the 40,342 patients scored, 2,613 (7%) were high risk and 29,417 (73%) were low risk in both scores. Only 2,613/6,771 (38.5%) of the patients rated as high risk by either approach were rated as high risk by both approaches, leading to a kappa of 0.26, or ‘fair’ agreement. This indicates that, even though we controlled for the differences of patients in the highest risk category for the practice and HCC (16%), the processes were mostly different. Comparisons by clinic are shown in Additional file 1.

The inside circle of Fig. 1 shows the subset of patients for whom outcomes were available and matched. Overall, 187, or 5% of the sample, was ranked high risk by both scores and 79% were ranked as not high risk, for an overall agreement of 84%. However, since only 5% of the sample was deemed high risk for both, the kappa agreement was fair (0.32).

Comparisons between HCC scores, practice-created scores, and utilization cut-off points for patients with reliable outcomes (88% Medicare patients, 12% dual eligible patients) show that HCC outperformed the practice stratification approach in the hospitalization and cost outcomes, but the approaches performed similarly in predicting frequent ED use (see Table 2). Accuracy was relatively high (0.71–0.88) but maximum achieved sensitivity (0.40) and PPV (0.34) were low. On average, the HCC score had higher absolute performance (2–14%) compared other stratification approaches, as shown in the difference (Practice-HCC).

**Table 2:** Selected diagnostic characteristics of practice-derived scores and HCC scores versus expenditure and utilization outcomes (N = 3,381)

Outcome	Practice: high risk	HCC: high risk	Δ (Practice-HCC)
	Average (range)	Average (range)	
ED ≥ 2 (12%)			
Sensitivity	0.3 (0.14, 0.69)	0.32 (0.17, 0.6)	− 0.02
PPV	0.25 (0.15, 0.33)	0.28 (0.23, 0.33)	− 0.03
Accuracy	0.76 (0.57, 0.87)	0.76 (0.53, 0.87)	0.00
Hospitalization ≥ 1 (14%)			
Sensitivity	0.26 (0.1, 0.53)	0.36 (0.16, 0.6)	− 0.10**
PPV	0.24 (0.15, 0.33)	0.34 (0.27, 0.5)	− 0.10**
Accuracy	0.74 (0.51, 0.84)	0.77 (0.54, 0.86)	− 0.03
Expenditures ≥ 30k (9%)			
Sensitivity	0.26 (0.07, 0.54)	0.4 (0.22, 0.66)	− 0.14**
PPV	0.16 (0.06, 0.32)	0.24 (0.19, 0.32)	− 0.08**
Accuracy	0.77 (0.51, 0.88)	0.79 (0.55, 0.9)	− 0.02

ED = Emergency Department use; PPV = Positive Predictive Value, or (True Positives)/(True Positives + False Positives); Sensitivity = True Positives/(True Positives + False Negatives); Accuracy = (True Positives + True Negatives)/All Cases; 30k = 30,000; HCC = Hierarchical Condition Category score

^*^p value < .05; **p value < .01; ***p value < .001

Table 3 shows that the practice using algorithm only as compared to algorithm plus adjudication consistently outperform their peers using clinical intuition for patients with higher utilization and cost. Scores from the four practices using adjudication had higher sensitivity (0.36–0.40) and Positive Predictive Value (PPV) (0.23–0.30) than the practice using clinical intuition (Sensitivity range 0.10–0.15; PPV range 0.06–0.17). The difference between practice and HCC was consistently smaller for *algorithm plus adjudication* than *clinical intuition* (− 0.09 to + 0.03 vs. − 0.21 to − 0.02; 0.157 average difference). Table 4 shows that practices with a worse (lower) perception of their risk stratification process consistently outperformed those with higher perception of the process for ED visits (Δ Practice − HCC Low − 0.02 to + 0.01 vs. High − 0.06 to − 0.03) and expenditures (Δ Practice − HCC Low − 0.09 to − 0.03 vs. High − 0.18 to − 0.03).

**Table 3:** Difference between practices using adjudication (N = 4, patients scored 2,088), clinical intuition on key metrics (N = 1, patients scored 359), or computer algorithm alone (N = 1, patients scored 934)

	Algorithm plus adjudication			Clinical intuition			Computer algorithm		
	Practice	HCC	Δ	Practice	HCC	Δ	Practice	HCC	Δ
ED ≥ 2 (12%)									
Sensitivity	0.40	0.37	0.03	0.15	0.33	− 0.18*	0.14	0.17	− 0.03
PPV	0.30	0.28	0.02	0.15	0.32	− 0.17*	0.19	0.23	− 0.04
Accuracy	0.72	0.70	0.02	0.78	0.82	− 0.04	0.87	0.87	0.00
Hospitalization ≥ 1 (14%)									
Sensitivity	0.36	0.44	− 0.09*	0.13	0.30	− 0.17*	0.10	0.16	− 0.06*
PPV	0.30	0.37	− 0.07*	0.15	0.34	− 0.19*	0.16	0.27	− 0.12*
Accuracy	0.70	0.73	− 0.03	0.76	0.81	− 0.05	0.84	0.86	− 0.02
Expenditures ≥ 30k (9%)									
Sensitivity	0.37	0.46	− 0.09*	0.14	0.41	− 0.27*	0.07	0.22	− 0.15**
PPV	0.23	0.27	− 0.04	0.06	0.19	− 0.13*	0.06	0.20	− 0.14**
Accuracy	0.71	0.74	− 0.03	0.82	0.86	− 0.04	0.88	0.90	− 0.02

ED = Emergency Department use; PPV = Positive Predictive Value; 30k = 30,000; HCC = Hierarchical Condition Category score

^*^p value < .05; **p value < .01; ***p value < .001

**Table 4:** Difference between practices with high (N = 4, patients scored 2,088) and low perception (N = 2, 1,293) of their risk stratification process

	High perception			Low perception		
	Practice	HCC	Δ	Practice	HCC	Δ
ED ≥ 2 (12%)						
Sensitivity	0.33	0.36	− 0.03	0.26	0.25	+ 0.01
PPV	0.21	0.27	− 0.06*	0.28	0.30	− 0.02
Accuracy	0.82	0.85	− 0.03	0.72	0.70	− 0.02
Hospitalization ≥ 1 (14%)						
Sensitivity	0.25	0.36	− 0.11**	0.27	0.36	− 0.09*
PPV	0.19	0.30	− 0.11**	0.32	0.41	− 0.09*
Accuracy	0.80	0.83	− 0.03	0.70	0.73	− 0.03
Expenditures ≥ 30k (9%)						
Sensitivity	0.25	0.43	− 0.18**	0.28	0.37	− 0.09*
PPV	0.11	0.22	− 0.11**	0.24	0.28	− 0.04
Accuracy	0.85	0.88	− 0.03	0.71	0.74	− 0.03

ED = Emergency Department use; PPV = Positive Predictive Value; 30k = 30,000; HCC = Hierarchical Condition Category score

^*^p value < .05; **p value < .01; ***p value < .001

## Discussion

We compared risk stratification generated from practice-based approaches versus a standard, automated approach. We also compared the accuracy of these approaches in predicting future utilization, and compared the accuracy of different practice-based risk stratification approaches with one another. Practices and HCC scores often identified different groups of patients as high risk: risk categories were discrepant in 20% of the sample. These differences can be partially explained by HCC scores, which focus on sets of chronic illnesses, such as diabetes, dementia, and heart disease, while the practices’ high risk patients had fewer of these conditions.

Overall performance was high for accuracy but low for sensitivity and PPV; the latter two are important: are you picking up most of at-risk patients (sensitivity)? and if you identify a patient as at-risk, how often are you correct (PPV)? The process used by the practices did not, in general, predict future outcomes more accurately or precisely, with up to 14 more patients/100 classified incorrectly by practices for future hospitalization and costs when using their score versus HCC scores. The differences in predicting ED visits were relatively minor. The approaches practices chose did affect prediction, with adjudication better than intuition and worse perception of the process better than a positive sense. Generally, the practices included in this study were smaller and more rural, leading to diverse approaches and perceptions. The improved performance with worse perception is somewhat puzzling; however, we have shown that this perception may be affected by desire to consistently improve the score. These results underscore the importance of provider and care team understanding computer-based algorithms. For instance, HCC algorithms may predict outcomes slightly better than practice chosen ones; however, adjudication may highlight other outcomes more proximal to the care team's approach [9].

These results are similar to others who explored clinical intuition based scoring versus algorithmic scores and the work of Hong et al. who showed that clinical adjudication of scores improved downstream prediction compared to algorithms or clinical intuition alone [11]. The fact that practice prediction lagged behind a standard algorithm may be due to the fact that these six practices were not part of large, integrated delivery systems (unlike Hong) and did not have advanced care management programs in place at the start of risk stratification. In addition, we’ve reported on the fact that data quality related to EHR use significantly impacts the value of the data [14], so providers’ intuition may have been impacted by sparse or inaccurate data more than the HCC algorithm. Additionally, the practices were required to care manage a majority of the patients they rated as highest risk, and if this approach was successful, it could have reduced the risk for adverse outcomes and expenditures and therefore the predictive validity. We did not use practices’ history of care management in the analysis because it was not tracked in a consistent manner and was judged equally likely to affect HCC predictive validity. We previously found that a human touch was important for uptake of the risk stratification approach [9] but, for these practices, pure clinical intuition as a risk stratification approach did not improve the ability to predict future outcomes; those using an algorithm consistently outperformed those who did not. The integration of clinical intuition and algorithm results to identify those at high risk is more complicated than this study shows; risks must be amenable to intervention.

Limitations of this study are the small number of practices, and variation in risk stratification approach, leading to challenges in comparisons. In addition, the errors were evenly balanced by design; we chose HCC cut-points which provided similar numbers of high risk patients as the practice set. Conversely, these differences may provide broader insights as to the variation possible in prediction of outcomes. The outcomes for the majority of the patients who were risk stratified were not known, making the analysis of risk prediction validity more limited. Confounding from risk mitigation strategies, like care management, may have also reduced our predictive validity since the practices may have acted immediately for high-risk patients, reducing their future utilization; however, these practices should affect both scores. Early results from the primary program most of the practices participated in, the Comprehensive Primary Care initiative, indicate that there was a reduction in utilization overall [15]. The source of this reduction is more likely from the patients the practices identified as high risk than those the HCC score identified, since the practices acted on the patients they identified; this was a source of potential endogeneity for which we could not adjust. Additionally, practices who agreed to participate from the hundreds (now thousands) completing risk stratification were likely biased. Large health systems were less likely to participate and those that did participate had to regularly record their scores. Practices who were not able to do this may have had even worse risk prediction processes.

## Conclusion

This work showed that algorithms were more accurate than clinical intuition in predicting unplanned utilization and cost; however, adjudication of those algorithm results demonstrated further improvement. This is highly relevant to population management in health care, as patients with complex conditions and multimorbidity represent the majority of health care utilization, yet predicting who will be high cost, high needs is a challenge for those working to mitigate these risks. Future research may consider combing the most accurate algorithms with adjudication and incorporate longer time windows with accurate information about how risk is managed. With a longer window, it may be easier to identify who is effectively care managed as their risk score may drop from year to year.

## Supplementary Information

**Additional file 1**. Confusion matrices by clinic

## Data Availability

The datasets generated and/or analyzed during the current study are not publicly available due to patient privacy regulations, but are available from the corresponding author on reasonable request.

## Abbreviations

DO: Doctor of osteopathy
ED: Emergency department
EHR: Electronic health record
FN: False negative
FP: False positive
HCC: Hierarchical condition category
HIT: Health information technology
MD: Medical doctor
NP: Nurse practitioner
PA: Physician assistant
PPV: Positive predictive value
RS: Risk stratification
TN: True negative
TP: True positive
