# Supplementary material for: Primary care practices’ ability to predict future risk of expenditures and hospitalization using risk stratification and segmentation
Source: BMC Med Inform Decis Mak. 2021 Mar 18;21:104. doi: 10.1186/s12911-021-01455-4 (PMC7977271; doi:10.1186/s12911-021-01455-4)
Supplement: Supplementary file 1 — Additional file 1. Confusion matrices by clinic [file 12911_2021_1455_MOESM1_ESM.docx]

Appendix 1. Confusion matrices by clinic

| \| \| **Frequency** \| \| --- \| \| **Percent** \| \| **Row Pct** \| \| **Col Pct** \| \| \| --- \| --- \| --- \| --- \| --- \| | \| **Table 1 of highhcc by highrisk** \| \| \| \| \| --- \| --- \| --- \| --- \| \| **Controlling for facility_id=1** \| \| \| \| \| **highhcc** \| **highrisk** \| \| \| \| **1. Yes** \| **2. No** \| **Total** \| \| **1. Yes** \| \| 32 \| \| --- \| \| 1.45 \| \| 12.08 \| \| 12.50 \| \| \| 233 \| \| --- \| \| 10.55 \| \| 87.92 \| \| 11.93 \| \| \| 265 \| \| --- \| \| 12.00 \| \|  \| \|  \| \| \| **2. No** \| \| 224 \| \| --- \| \| 10.14 \| \| 11.52 \| \| 87.50 \| \| \| 1720 \| \| --- \| \| 77.86 \| \| 88.48 \| \| 88.07 \| \| \| 1944 \| \| --- \| \| 88.00 \| \|  \| \|  \| \| \| **Total** \| \| 256 \| \| --- \| \| 11.59 \| \| \| 1953 \| \| --- \| \| 88.41 \| \| \| 2209 \| \| --- \| \| 100.00 \| \| |
| --- | --- | --- | --- | --- | --- | --- | --- | --- | --- | --- | --- | --- | --- | --- | --- | --- | --- | --- | --- | --- | --- | --- | --- | --- | --- | --- | --- | --- | --- | --- | --- | --- | --- | --- | --- | --- | --- | --- | --- | --- | --- | --- | --- | --- | --- | --- | --- | --- | --- | --- | --- | --- | --- | --- | --- | --- | --- | --- | --- | --- | --- | --- | --- |

| \| \| **Frequency** \| \| --- \| \| **Percent** \| \| **Row Pct** \| \| **Col Pct** \| \| \| --- \| --- \| --- \| --- \| --- \| | \| **Table 2 of highhcc by highrisk** \| \| \| \| \| --- \| --- \| --- \| --- \| \| **Controlling for facility_id=2** \| \| \| \| \| **highhcc** \| **highrisk** \| \| \| \| **1. Yes** \| **2. No** \| **Total** \| \| **1. Yes** \| \| 87 \| \| --- \| \| 1.36 \| \| 19.55 \| \| 19.51 \| \| \| 358 \| \| --- \| \| 5.61 \| \| 80.45 \| \| 6.03 \| \| \| 445 \| \| --- \| \| 6.97 \| \|  \| \|  \| \| \| **2. No** \| \| 359 \| \| --- \| \| 5.63 \| \| 6.05 \| \| 80.49 \| \| \| 5576 \| \| --- \| \| 87.40 \| \| 93.95 \| \| 93.97 \| \| \| 5935 \| \| --- \| \| 93.03 \| \|  \| \|  \| \| \| **Total** \| \| 446 \| \| --- \| \| 6.99 \| \| \| 5934 \| \| --- \| \| 93.01 \| \| \| 6380 \| \| --- \| \| 100.00 \| \| |
| --- | --- | --- | --- | --- | --- | --- | --- | --- | --- | --- | --- | --- | --- | --- | --- | --- | --- | --- | --- | --- | --- | --- | --- | --- | --- | --- | --- | --- | --- | --- | --- | --- | --- | --- | --- | --- | --- | --- | --- | --- | --- | --- | --- | --- | --- | --- | --- | --- | --- | --- | --- | --- | --- | --- | --- | --- | --- | --- | --- | --- | --- | --- | --- |

| \| \| **Frequency** \| \| --- \| \| **Percent** \| \| **Row Pct** \| \| **Col Pct** \| \| \| --- \| --- \| --- \| --- \| --- \| | \| **Table 3 of highhcc by highrisk** \| \| \| \| \| --- \| --- \| --- \| --- \| \| **Controlling for facility_id=3** \| \| \| \| \| **highhcc** \| **highrisk** \| \| \| \| **1. Yes** \| **2. No** \| **Total** \| \| **1. Yes** \| \| 339 \| \| --- \| \| 12.95 \| \| 43.30 \| \| 43.35 \| \| \| 444 \| \| --- \| \| 16.97 \| \| 56.70 \| \| 24.20 \| \| \| 783 \| \| --- \| \| 29.92 \| \|  \| \|  \| \| \| **2. No** \| \| 443 \| \| --- \| \| 16.93 \| \| 24.15 \| \| 56.65 \| \| \| 1391 \| \| --- \| \| 53.15 \| \| 75.85 \| \| 75.80 \| \| \| 1834 \| \| --- \| \| 70.08 \| \|  \| \|  \| \| \| **Total** \| \| 782 \| \| --- \| \| 29.88 \| \| \| 1835 \| \| --- \| \| 70.12 \| \| \| 2617 \| \| --- \| \| 100.00 \| \| |
| --- | --- | --- | --- | --- | --- | --- | --- | --- | --- | --- | --- | --- | --- | --- | --- | --- | --- | --- | --- | --- | --- | --- | --- | --- | --- | --- | --- | --- | --- | --- | --- | --- | --- | --- | --- | --- | --- | --- | --- | --- | --- | --- | --- | --- | --- | --- | --- | --- | --- | --- | --- | --- | --- | --- | --- | --- | --- | --- | --- | --- | --- | --- | --- |

| \| \| **Frequency** \| \| --- \| \| **Percent** \| \| **Row Pct** \| \| **Col Pct** \| \| \| --- \| --- \| --- \| --- \| --- \| | \| **Table 4 of highhcc by highrisk** \| \| \| \| \| --- \| --- \| --- \| --- \| \| **Controlling for facility_id=4** \| \| \| \| \| **highhcc** \| **highrisk** \| \| \| \| **1. Yes** \| **2. No** \| **Total** \| \| **1. Yes** \| \| 934 \| \| --- \| \| 3.86 \| \| 29.68 \| \| 29.49 \| \| \| 2213 \| \| --- \| \| 9.15 \| \| 70.32 \| \| 10.53 \| \| \| 3147 \| \| --- \| \| 13.01 \| \|  \| \|  \| \| \| **2. No** \| \| 2233 \| \| --- \| \| 9.23 \| \| 10.61 \| \| 70.51 \| \| \| 18812 \| \| --- \| \| 77.76 \| \| 89.39 \| \| 89.47 \| \| \| 21045 \| \| --- \| \| 86.99 \| \|  \| \|  \| \| \| **Total** \| \| 3167 \| \| --- \| \| 13.09 \| \| \| 21025 \| \| --- \| \| 86.91 \| \| \| 24192 \| \| --- \| \| 100.00 \| \| |
| --- | --- | --- | --- | --- | --- | --- | --- | --- | --- | --- | --- | --- | --- | --- | --- | --- | --- | --- | --- | --- | --- | --- | --- | --- | --- | --- | --- | --- | --- | --- | --- | --- | --- | --- | --- | --- | --- | --- | --- | --- | --- | --- | --- | --- | --- | --- | --- | --- | --- | --- | --- | --- | --- | --- | --- | --- | --- | --- | --- | --- | --- | --- | --- |

| \| \| **Frequency** \| \| --- \| \| **Percent** \| \| **Row Pct** \| \| **Col Pct** \| \| \| --- \| --- \| --- \| --- \| --- \| | \| **Table 5 of highhcc by highrisk** \| \| \| \| \| --- \| --- \| --- \| --- \| \| **Controlling for facility_id=5** \| \| \| \| \| **highhcc** \| **highrisk** \| \| \| \| **1. Yes** \| **2. No** \| **Total** \| \| **1. Yes** \| \| 829 \| \| --- \| \| 23.20 \| \| 59.68 \| \| 55.12 \| \| \| 560 \| \| --- \| \| 15.67 \| \| 40.32 \| \| 27.05 \| \| \| 1389 \| \| --- \| \| 38.86 \| \|  \| \|  \| \| \| **2. No** \| \| 675 \| \| --- \| \| 18.89 \| \| 30.89 \| \| 44.88 \| \| \| 1510 \| \| --- \| \| 42.25 \| \| 69.11 \| \| 72.95 \| \| \| 2185 \| \| --- \| \| 61.14 \| \|  \| \|  \| \| \| **Total** \| \| 1504 \| \| --- \| \| 42.08 \| \| \| 2070 \| \| --- \| \| 57.92 \| \| \| 3574 \| \| --- \| \| 100.00 \| \| |
| --- | --- | --- | --- | --- | --- | --- | --- | --- | --- | --- | --- | --- | --- | --- | --- | --- | --- | --- | --- | --- | --- | --- | --- | --- | --- | --- | --- | --- | --- | --- | --- | --- | --- | --- | --- | --- | --- | --- | --- | --- | --- | --- | --- | --- | --- | --- | --- | --- | --- | --- | --- | --- | --- | --- | --- | --- | --- | --- | --- | --- | --- | --- | --- |

| \| \| **Frequency** \| \| --- \| \| **Percent** \| \| **Row Pct** \| \| **Col Pct** \| \| \| --- \| --- \| --- \| --- \| --- \| | \| **Table 6 of highhcc by highrisk** \| \| \| \| \| --- \| --- \| --- \| --- \| \| **Controlling for facility_id=6** \| \| \| \| \| **highhcc** \| **highrisk** \| \| \| \| **1. Yes** \| **2. No** \| **Total** \| \| **1. Yes** \| \| 392 \| \| --- \| \| 28.61 \| \| 52.83 \| \| 64.05 \| \| \| 350 \| \| --- \| \| 25.55 \| \| 47.17 \| \| 46.17 \| \| \| 742 \| \| --- \| \| 54.16 \| \|  \| \|  \| \| \| **2. No** \| \| 220 \| \| --- \| \| 16.06 \| \| 35.03 \| \| 35.95 \| \| \| 408 \| \| --- \| \| 29.78 \| \| 64.97 \| \| 53.83 \| \| \| 628 \| \| --- \| \| 45.84 \| \|  \| \|  \| \| \| **Total** \| \| 612 \| \| --- \| \| 44.67 \| \| \| 758 \| \| --- \| \| 55.33 \| \| \| 1370 \| \| --- \| \| 100.00 \| \| |
| --- | --- | --- | --- | --- | --- | --- | --- | --- | --- | --- | --- | --- | --- | --- | --- | --- | --- | --- | --- | --- | --- | --- | --- | --- | --- | --- | --- | --- | --- | --- | --- | --- | --- | --- | --- | --- | --- | --- | --- | --- | --- | --- | --- | --- | --- | --- | --- | --- | --- | --- | --- | --- | --- | --- | --- | --- | --- | --- | --- | --- | --- | --- | --- |
